# Improvement of bone mineral density after enzyme replacement therapy in Chinese late-onset Pompe disease patients

**DOI:** 10.1186/s13104-017-2681-y

**Published:** 2017-07-28

**Authors:** Bun Sheng, Yim Pui Chu, Wa Tai Wong, Eric Kin Cheong Yau, Sammy Pak Lam Chen, Wing Hang Luk

**Affiliations:** 1 Department of Medicine & Geriatrics, Princess Margaret Hospital, Lai Chi Kok, Kowloon, Hong Kong SAR; 2 Department of Paediatrics and Adolescent Medicine, Princess Margaret Hospital, Kowloon, Hong Kong SAR; 3 Department of Pathology, Princess Margaret Hospital, Kowloon, Hong Kong SAR; 4 Department of Radiology, Princess Margaret Hospital, Kowloon, Hong Kong SAR

**Keywords:** Glycogen storage disease type II, Lysosomal storage diseases, Osteoporosis, Enzyme replacement therapy, Bone density, Body weight

## Abstract

**Objective:**

Late-onset Pompe disease (LOPD) is a lysosomal storage disease resulted from deficiency of the enzyme acid α-glucosidase. Patients usually develop a limb-girdle pattern of myopathy and respiratory impairment, and enzyme replacement therapy (ERT) is the only specific treatment available. Recently, LOPD has been associated with low bone mineral density (BMD), but the effect of ERT on BMD is inconclusive. In this report we described our early observations on the change of BMD after ERT in Chinese LOPD patients.

**Results:**

We studied four Chinese LOPD patients with different severities of myopathy. All were underweight, and three had osteoporosis at baseline. We found significant weight gain in three patients after ERT and all four patients showed improvement in BMD. The biggest improvement, 84.4% increase in BMD, was seen in a lady with the most prominent weight recovery. Our results suggest that ERT improves BMD in Chinese LOPD and weight gain could be a major contributor to this effect.

## Introduction

Pompe disease is an autosomal recessive disorder caused by the deficiency of a lysosomal enzyme, acid α-glucosidase, resulting in glycogen accumulations and consequent autophagic buildup [1–3]. The late-onset form (LOPD) is characterized by a limb-girdle pattern of myopathy and respiratory impairment [4]. At present, enzyme replacement therapy (ERT) with alglucosidase alfa is the only specific treatment available. It modestly improves mobility and stabilizes respiratory function in patients with LOPD [5].

Recently, low bone mineral density (BMD) and osteoporosis has been reported in LOPD [6, 7]. These, and together with the observations on many other non-muscle disease manifestations such as small-fiber neuropathy, torturous vessels, minor cardiac abnormalities etc., have redefined LOPD as a multi-system disease [8, 9]. At the moment, it is unclear whether these non-myopathy parameters would be modified by ERT. We examined six patients with dual X-ray absorptiometry (DXA) upon initiation of ERT and found that three had osteoporosis and one had osteopenia. Herein, we present our observations on the first four patients who completed the follow-up DXA study.

## Main text

### Methods

Four Chinese LOPD patients who underwent DXA (Prodigy Advance, GE Healthcare) at baseline before ERT and a follow-up study after ERT were included. Patients 1 and 2 were brothers, patients 3 and patient 4 were sisters. Patient 1 was a juvenile-onset patient with an aggressive disease course; he was wheelchair-bound and required full-day non-invasive ventilation (NIV) support upon initiation of ERT. Because of his previous spinal surgery for scoliosis, patient 1’s DXA was performed on the forearm and hip instead of the usual lumbar spine and hip. Patient 2 was fully ambulatory and did not require NIV. Both patient 3 and patient 4 presented with type II respiratory failure in their early 30s. They were ambulatory and on nocturnal NIV. We prescribed calcium, vitamin D and l-alanine supplements to all of our LOPD patients. Patient 3 and patient 4 also received alendronate for osteoporosis after the baseline DXA study. Alglucosidase alfa infusion was given at the standard regime of 20 mg/kg every 2 weeks.

We serially monitored the patients’ mobility and pulmonary function with the 6-min walk test (6MWT) and spirometry, respectively, according to our internal management protocol for all LOPD patients on ERT, in order to justify the continuation of ERT in these patients through public funding. We also used a modified Walton scale according to Slonim et al. (0: all activities normal, to 7: wheelchair bound) to assess the muscle weakness in relation to their daily functions [10]. A follow-up DXA was arranged for patients 1, 2 and 3 after 5 years of ERT. The DXA was scheduled earlier for patient 4 because of her significant weight gain since the ERT.

### Results

The four patients were in different stages of severity across the disease spectrum (Table 1). They were all slim and underweight. BMD measurements at baseline revealed that patients 1, 3 and 4 had osteoporosis, while patient 2 was in the normal range. All four patients had significant respiratory impairment before ERT as measured by spirometry, though patient 2 did not require NIV.

**Table 1:** Baseline characteristics of patients at study entry before enzyme replacement therapy

	Genotype	Age	Gender	Walton score	Assisted ventilation
Patient 1	c.1082C>T (p.Pro361Leu) c.1309C>T (p.Arg437Cys)	25	M	7	Full day NIV
Patient 2	c.1082C>T (p.Pro361Leu) c.1309C>T (p.Arg437Cys)	21	M	2	Nil
Patient 3	c.2238G>C (p.Trp746Cys) c.1935C>A (p.Asp645Glu)	39	F	2	Nocturnal NIV
Patient 4	c.2238G>C (p.Trp746Cys) c.1935C>A (p.Asp645Glu)	37	F	2.5	Nocturnal NIV

Patients 1 and 2 were brothers, patients 3 and 4 were sisters

**Fig. 1:**
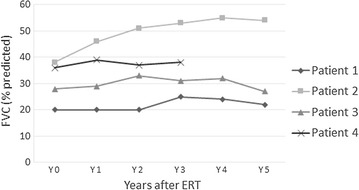
Serial changes in FVC after ERT

**Fig. 2:**
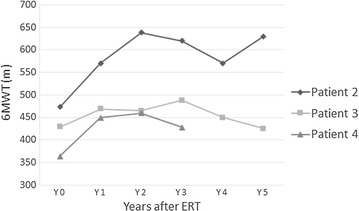
Serial changes in 6MWT (m) after ERT

In the follow-up reassessment after ERT (Figs. 1, 2; Table 2), patient 2 showed significant and sustained improvement in mobility and pulmonary function. Pulmonary function in patients 1 and 4 had improved slightly, and the effect was maintained over the study period. Patient 4 also showed better mobility and physical endurance; her Walton score decreased from 2.5 to 2. Patient 3 had initial mild improvement in mobility and pulmonary function, but both parameters dropped to baseline levels later. None of the four patients showed any change in requirement or degree of NIV support, and except patient 4, their respective Walton scores also remained the same.

**Table 2:** Changes in mobility, pulmonary function, body mass index and bone mineral density after enzyme replacement therapy at the time of follow-up DXA

	Duration of ERT (months)	6MWT (m)		Walton score		FVC (L) (% predicted)		BMI (kg/m^2^)		BMD (g/cm^2^)			∆BMD (%)
		Pre-ERT	Post-ERT	Pre-ERT	Post-ERT	Pre-ERT	Post-ERT	Pre-ERT	Post-ERT		Pre-ERT	Post-ERT	
Patient 1	62	NA	NA	7	7	0.94 (20%)	1.02 (22%)	14.2	17.0	33% radius^a^	0.765	0.825	+7.8
										Z-score	−1.9	−1.3	
										Hip total	0.675	0.749	+11
										Z-score	−2.5	−1.9	
Patient 2	61	473	630	2	2	1.63 (38%)	2.32 (54%)	16.2	19.6	Spine L1–L4	1.209	1.328	+9.8
										Z-score	0.9	2.0	
										Hip total	0.928	0.939	+1.2
										Z-score	−0.5	−0.4	
Patient 3	61	429	425	2	2	0.74 (28%)	0.70 (27%)	14.3	13.6	Spine L1–L4	0.959	1.027	+7.1
										Z-score	−1.3	−0.7	
										Hip total	0.576	0.642	+11.5
										Z-score	−3.1	−2.4	
Patient 4	33	364	428	2.5	2	0.97 (36%)	0.99 (38%)	16.2	20.6	Spine L1–L4	0.940	1.341	+42.7
										Z-score	−1.5	1.9	
										Hip total	0.41	0.773	+84.4
										Z-score	−4.3	−1.5	

Patients 1 and 2 were brothers, patients 3 and 4 were sisters

*FVC* forced vital capacity, *BMI* body mass index, *NA* not applicable

^a^Patient 1 had spinal surgery for scoliosis, BMD was measured from forearm instead of lumbar vertebra

Significant weight gains of 22, 21 and 27% was observed in patients 1, 2 and 4, respectively. Patient 3 did not gain weight after ERT and remained severely underweight. BMD had increased in all four patients after ERT. There was an increase of approximately 10% in patients 1, 2 and 3 and an astonishing 84.4% at the hip in patient 4. The improved Z-scores showed that patients 1, 3 and 4 had moved from osteoporosis to osteopenia after ERT.

### Discussion

Low BMD causing a predisposition to fracture has been associated with LOPD [11, 12], but publications specifically addressing this are lacking. The only systematic study that involved 46 Pompe patients (both late- and infantile-onset forms) reported osteoporosis in 26%, and the majority of the affected were older, wheelchair-bound patients with long disease duration, leading to the speculation that weak loading force had led to osteoporosis [13]. Similar observations have been reported for other chronic myopathies, and the speculation was supported by an experimental study applying computed tomography to analyze the bone architecture [14, 15]. However, osteoporosis appears to be over-represented in LOPD compared to other severe hereditary myopathies such as Duchenne muscular dystrophy, in which most patients are wheelchair-bound as teenagers, while the majority of the LOPD patients are still ambulatory. Muscle strength per se is not a sufficient explanation for osteoporosis in LOPD. Chinese LOPD patients are characterized by an aggressive disease course with earlier emergence of symptoms, rapid deterioration and early respiratory failure, and most of them are slim and underweight [16, 17]. Since malnutrition and low body weight are major risk factors for low peak bone mass and BMD, this Chinese LOPD phenotype could be particularly vulnerable to osteoporosis. Our DXA results from a small sample of six patients with this typical phenotype do suggest that osteoporosis is more prevalent in Chinese LOPD. We believe that any BMD changes caused by ERT could be more readily observable in these high-risk patients.

Our four patients represented a spectrum of different disease severities, but they were all underweight at baseline before ERT. Three had osteoporosis, and one had normal BMD. All four patients showed improvement in BMD in the follow-up study. Patient 4 had the most prominent weigh gain, and her physical endurance improved, but there was no significant enhancement in her hip girdle muscle strength, and her mobility was only modestly better. Her huge gain in BMD was more likely a result of her recovery of body weight. Alendronate could not improve BMD by that much, and any positive effect from improvement in muscle strength should be modest. Patients 1 and 2 were both underweight and had similar weight gain after ERT. Patient 1 remained severely disabled, and patient 2 had good improvement in physical performance. The improvement in BMD was more marked in patient 1 than in patient 2, suggesting that the treatment effect could be more obvious in those with a lower baseline BMD and that strength might be less important than body weight. These observations agreed with the previous reports that described inconsistent ERT responses in LOPD patients with diverse characteristics [6, 18]. Nevertheless, patient 3 had similar disease severity to patient 4, but she did not gain weight, and her physical performance deteriorated slightly in the follow-up, yet she still had a 10% increase in BMD. Enzyme therapy could have a direct positive effect on bone turnover independent of weight and muscle strength.

Although it is acknowledged that Chinese LOPD patients commonly manifest an aggressive clinical course and low BMI is a poor prognostic marker in LOPD, the reason for such a high prevalence of malnutrition in Chinese LOPD is still poorly understood. Postulations include, among many, a chronic malabsorption state from gastrointestinal smooth muscle involvement in LOPD and a persistent catabolic state from respiratory failure [19, 20]. However, neither would be a strong contender with an impact on nutritional status to the degree we observe in LOPD.

## Limitations

We observed a positive effect on BMD after ERT in a small group of Chinese LOPD patients, and suggested a few postulations to explain this association. However, our findings were mainly hypothesis generating, we were unable establish any causal relationship between ERT and BMD. Future studies should focus on catabolism, changes in body compositions, changes in bone turnover markers, and the balance of hormones and cytokines, both at baseline and with ERT, and take the secondary contributing factors into account to obtain a clearer picture on LOPD and bone health.

## Abbreviations

6MWT: 6-min walk test
BMD: bone mineral density
BMI: body mass index
DXA: dual X-ray absorptiometry
ERT: enzyme replacement therapy
FVC: forced vital capacity
LOPD: late-onset Pompe disease
NIV: non-invasive ventilation
